# Allosteric Antagonism of the A_2A_ Adenosine Receptor by a Series of Bitopic Ligands

**DOI:** 10.3390/cells9051200

**Published:** 2020-05-12

**Authors:** Zhan-Guo Gao, Kiran S. Toti, Ryan Campbell, R. Rama Suresh, Huijun Yang, Kenneth A. Jacobson

**Affiliations:** Molecular Recognition Section, Laboratory of Bioorganic Chemistry, National Institute of Diabetes and Digestive and Kidney Diseases, National Institutes of Health, Bethesda, MD 20892, USA; kiran.toti@nih.gov (K.S.T.); rcampbell19@wooster.edu (R.C.); raavi.hcu@gmail.com (R.R.S.); lwyhj78@yahoo.com (H.Y.)

**Keywords:** allosteric modulation, GPCR, bitopic ligands, A_2A_ adenosine receptor, antagonist

## Abstract

Allosteric antagonism by bitopic ligands, as reported for many receptors, is a distinct modulatory mechanism. Although several bitopic A_2A_ adenosine receptor (A_2A_AR) ligand classes were reported as pharmacological tools, their receptor binding and functional antagonism patterns, i.e., allosteric or competitive, were not well characterized. Therefore, here we systematically characterized A_2A_AR binding and functional antagonism of two distinct antagonist chemical classes. i.e., fluorescent conjugates of xanthine amine congener (XAC) and SCH442416. Bitopic ligands were potent, weak, competitive or allosteric, based on the combination of pharmacophore, linker and fluorophore. Among antagonists tested, XAC, XAC245, XAC488, SCH442416, MRS7352 showed K_i_ binding values consistent with K_B_ values from functional antagonism. Interestingly, MRS7396, XAC-X-BY630 (XAC630) and 5-(*N*,*N*-hexamethylene)amiloride (HMA) were 9–100 times weaker in displacing fluorescent MRS7416 binding than radioligand binding. XAC245, XAC630, MRS7396, MRS7416 and MRS7322 behaved as allosteric A_2A_AR antagonists, whereas XAC488 and MRS7395 antagonized competitively. Schild analysis showed antagonism slopes of 0.42 and 0.47 for MRS7396 and XAC630, respectively. Allosteric antagonists HMA and MRS7396 were more potent in displacing [^3^H]ZM241385 binding than MRS7416 binding. Sodium site D52N mutation increased and decreased affinity of HMA and MRS7396, respectively, suggesting possible preference for different A_2A_AR conformations. The allosteric binding properties of some bitopic ligands were rationalized and analyzed using the Hall two-state allosteric model. Thus, fluorophore tethering to an orthosteric ligand is not neutral pharmacologically and may confer unexpected properties to the conjugate.

## 1. Introduction

The A_2A_ adenosine receptor (A_2A_AR), a major target of caffeine, is an attractive drug target for many conditions. Both A_2A_AR agonists and antagonists have been approved for clinical use [1,2,3]. For example, the agonist Regadenoson is being widely used for cardiac stress test. The xanthine antagonist istradefylline has been approved as a cotherapy for treating Parkinson’s disease. Despite the tremendous therapeutic opportunities associated with important A_2A_AR physiological functions, most A_2A_AR ligand clinical trials have failed [1,2], which is due at least in part to the lack of understanding of mechanisms related to receptor binding and activation, which is relevant to both therapeutic and side effects. Further understanding of its A_2A_AR function and regulation, especially its allosteric modulation could provide an important basis for development of agonists, antagonists and allosteric modulators of the A_2A_AR with better therapeutic effects and fewer side effects.

A_2A_AR allosteric modulation by amiloride analogs and sodium ions has been previously reported [4]. The high-resolution structure of the A_2A_AR with a sodium ion bound shows exquisite detail [5], and its sodium modulation has been extensively explored using both NMR and X-ray techniques [6,7,8]. Amiloride analogs have been suggested to bind to the sodium binding pocket, showing distinct patterns of modulation of agonist and antagonist binding [9,10,11]. In addition to sodium ions and amilorides, Sun et al. [12] crystallized the A_2A_AR complex with a structurally novel bitopic antagonist, Compound **1**, which occupies the orthosteric site for adenosine and extends into an adjacent binding pocket toward the extracellular portions of transmembrane helical domain 1 (TM1), TM2 and TM7. The adjacent pocket pointing to the extracellular part of TMs was proposed as a novel allosteric binding site, and the flexibility in the binding pockets was emphasized [12].

**Figure 1:**
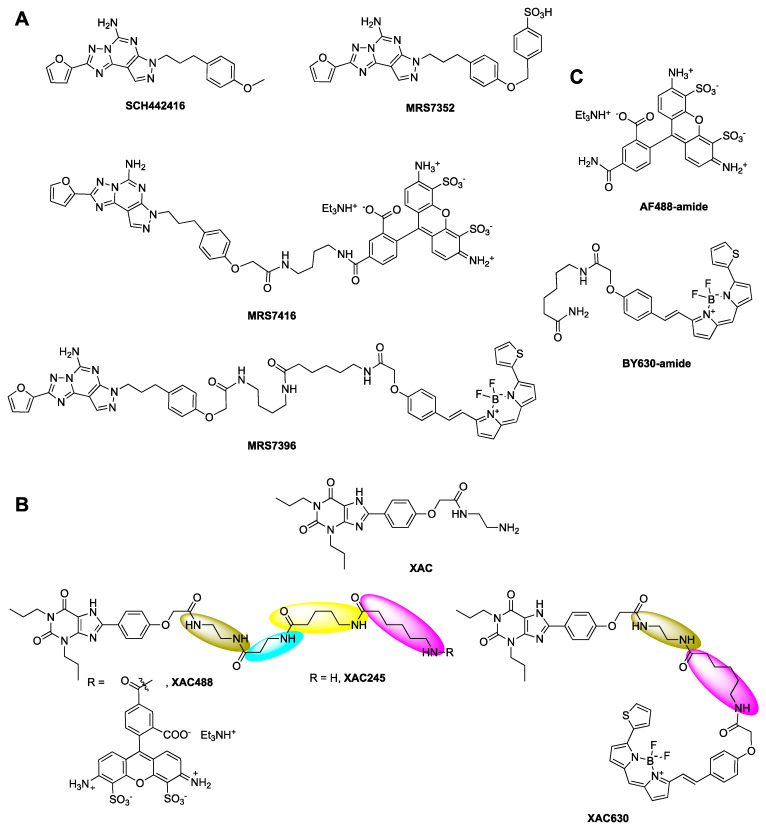
Chemical structures of ligands used in the present study. (**A**). Derivatives of SCH442416. (**B**). Derivatives of xanthine amine congener (XAC). (**C**). Amide reference derivatives of the fluorophores AF488 and Bodipy-630. Structures of MRS7322 and MRS7395 are given in Duroux et al. [18]. XAC630 is also known as XAC-X-BY630, CA200634 and A-633-AN [27].

Fluorescent ligands are useful tools to study ligand-receptor binding, receptor polarization, expression, kinetics and real-time monitoring of living cells, and have been developed for all four AR subtypes [13,14,15,16]. We have previously developed selective A_2A_AR fluorescent ligands based on functionalized congeners [17] of pyrazolotriazolopyrimidine antagonist SCH442416 labeled with an AlexaFluor488 dye, which have been used successfully for fluorescence polarization and flow cytometry assays [16,18]. A boron-dipyrromethene (Bodipy) 630/650-labeled conjugate of xanthine amine congener (XAC, [17]) has been used for A_1_AR quantification [15]. However, the A_2A_AR binding patterns of these ligands have not been extensively characterized. It is not clear if A_2A_AR antagonism by bitopic ligands is allosteric or orthosteric in nature, although allosteric modulation by bitopic ligands has been reported for many other G protein-coupled receptors (GPCRs) [19,20,21,22,23,24,25]. Therefore, in the present study, we systematically characterized the patterns of binding and functional antagonism at the A_2A_AR of two distinct classes of A_2A_AR antagonists conjugated with different moieties, based on xanthine XAC and nonxanthine SCH442416 (Figure 1). It was found that the bitopic ligands could be potent, weak, competitive or allosteric, which was dependent on the combination of A_2A_AR antagonist pharmacophore, linker and fluorophore. The allosteric binding properties of some bitopic ligands were rationalized and analyzed with a mathematical model based on the two-state allosteric model of allosteric modulation [26].

## 2. Materials and Methods

### 2.1. Materials

[^3^H]-2-[p-(2-Carboxyethyl)phenylethylamino]-5′-*N*-ethylcarboxamido-adenosine ([^3^H]CGS21680, 30.5 Ci/mmol) was purchased from PerkinElmer (Waltham, MA, USA). [^3^H]4-[2-[7-amino-2-(2-furyl)-1,2,4-triazolo [1,5-*a*][1,3,5]triazin-5-yl-amino]ethylphenol ([^3^H]ZM241385; 50 Ci/mmol) was from American Radiolabeled Chemicals (St. Louis, MO, USA). DMEM medium and 1 M Tris–HCl (pH 7.5) were purchased from Mediatech, Inc. (Herndon, VA, USA). Adenosine deaminase was from Worthington Biochemical Corp. (Lakewood, NJ, USA). AR ligands 9-chloro-2-(2-furanyl)-[1,2,4]triazolo[1,5-*c*]quinazolin-5-amine (CGS15943), CGS21680, 2-(2-Furanyl)-7-[3-(4-methoxyphenyl)propyl]-7H-pyrazolo[4,3-*e*][1,2,4]triazolo[1,5-*c*]pyrimidin-5-amine (SCH442416), ZM241385 and 2-amino-4-(4-hydroxyphenyl)-6-[(1*H*-imidazol-2-ylmethyl)thio]-3,5-pyridinecarbonitrile (LUF5834) were from Tocris (Ellisville, MO, USA). XAC-X-BY630 (CA200634 CellAura fluorescent adenosine antagonist, termed XAC630 in this work) was purchased from Hello Bio, Inc. (Princeton, NJ 08540, USA). XAC, XAC245 (synthesis in Supporting information, amine congener from which is a fragment of a different Bodipy conjugate CA200645, containing an extra β-Ala-5-amino-valeryl linker compared to XAC630) [27], Bodipy-630-amide (BY630-amide), AlexaFluor-488-amide (AF488-amide), MRS7416 and MRS7396 (structures in Figure 1) were synthesized at NIDDK, National Institutes of Health (Bethesda, MD, USA). UK432097 was obtained from Sigma-Aldrich (St. Louis, MO, USA). All other materials were from standard commercial sources and of analytical grade.

### 2.2. Cell Culture, Membrane Preparation and Radioligand Binding

HEK293 cells expressing the human A_2A_AR were cultured in DMEM supplemented with 10% fetal bovine serum, 100 Units/mL penicillin, 100 µg/mL streptomycin and 2 µmol/mL glutamine. To prepare cell membranes, cells were detached from plates by scraping into PBS and centrifuged at 250× *g* for 5 min. The resulting pellets were re-suspended in ice-cold Tris-HCl buffer (50 mM, pH 7.4) and homogenized. After homogenization and suspension, cells were first centrifuged at 1000× *g* for 10 min and the pellet was discarded. The suspension was then re-centrifuged at 20,000× *g* for 60 min at 4 °C. The pellets were re-suspended, split into aliquots and stored at −80 °C. The protein concentration was measured using the Bradford assay.

Radioligand binding assays were performed using membranes prepared from HEK293 cells expressing human the A_2A_AR using both agonist and antagonist radioligands as previously described [4,6,8]. For ligand displacement experiments, increasing concentrations of ligands were incubated with [^3^H]CGS21680 (5 nM) or [^3^H]ZM241385 (1 nM) and membrane preparations (20 μg protein) at 25 °C for 60 min in a total of 200 μL of Tris-HCl buffer (50 mM, pH 7.5) containing 10 mM MgCl_2_. XAC (10 μM) was used to determine nonspecific binding. Binding reactions were terminated by filtration through Whatman GF/B filters under reduced pressure using an MT-24 cell harvester (Brandell, Gaithersburg, MD, USA) followed by washing twice with 5 mL of cold Tris-HCl buffer. Radioactivity was measured using a scintillation counter (Tri-Carb 2810TR).

### 2.3. Fluorescent Ligand Binding to Intact HEK293 Cells Expressing the Human A_2A_AR

Cells were plated in 96-well plates and grow overnight. In saturation binding experiments with fluorescent antagonist MRS7416, various concentrations were incubated in cell culture medium with control HEK293 cells or HEK293 cells expressing the A_2A_AR for 60 min at 37 °C in an atmosphere containing 5% CO_2_. Nonspecific binding was determined using XAC (10 μM). To determine binding affinities of A_2A_AR ligands using MRS7416 as a tracer, A_2A_AR-expressing HEK293 cells were incubated with different concentrations of ligands in the presence of 15 nM MRS7416 for 60 min. At the end the reaction, the medium was removed, and cells were washed twice with PBS. After washing, 0.2 mL 0.1% EDTA solution was added to each well, and cells were incubated at 37 °C for 10 min. Following cell detachment and mixing of cells, the intensity of fluorescence emission of each sample was measured by using flow cytometry as previously described [18]. Cell suspensions were vortexed briefly before analysis on a Becton and Dickinson FACS Calibur flow cytometer (BD, Franklin Lakes, NJ, USA) with excitation at 488 nm. Samples were maintained in the dark during the analysis to avoid photobleaching. Mean fluorescence intensity (MFI) was determined in the FL-1 channel in log mode. Ten thousand events were analyzed per sample. Data were collected using Cell Quest Pro software (BD, Franklin Lakes, NJ, USA) and analyzed by Cyflogic v. 1.2.1 software (CyFlo, Ltd., Turku, Finland).

### 2.4. cAMP Assay

HEK293 cells stably expressing the human A_2A_AR were seeded in 96-well plates and incubated in 100 µL medium at 37 °C overnight. The medium was removed the following day and cells were then treated with assay buffer containing rolipram (10 μM) and antagonists for 20 min followed by the addition of agonists, and then incubated for 20 min. The reaction was terminated upon removal of the supernatant and addition of 100 µL Tween-20 (0.3%). Intracellular 3′,5′-cyclic adenosine monophosphate (cAMP) levels were measured with an ALPHAScreen cAMP assay kit as instructed by the manufacturer (PerkinElmer, Boston, MA, USA).

### 2.5. Simulation of Concentration–Effect Curves Using Wolfram Mathematica

A mathematical model for allosteric modulation [26] was implemented in Wolfram Mathematica (Champaign, IL, USA, version 11.3), a computational software package.

### 2.6. Statistical Analysis

Functional parameters were calculated using Prism 8.1.0 software (GraphPAD, San Diego, CA, USA). Data were expressed as mean ± standard error. Data were analyzed by analysis of variance (ANOVA) followed by post hoc analysis to check the statistical difference among groups with *p* < 0.05 being considered significant. For radioligand binding, calculation of apparent binding affinities, with K_i_ = IC_50_/(1 + [radioligand]/K_d_), where K_i_ is the inhibition constant and K_d_ the equilibrium dissociation constant, was performed using the nonlinear iterative curve-fitting program of Prism (GraphPad Software Inc., San Diego, CA, USA).

### 2.7. Chemical Synthesis

Materials and Methods. AlexaFluor488-5TFP ester was purchased from Thermo Fisher Scientific (New York, NY, USA). BODIPY630/650-NHS was purchased from Lumiprobe (Cockeysville, MD, USA). All other chemicals and solvents were from Sigma-Aldrich (St. Louis, MO, USA). Anhydrous solvents were obtained directly from commercial sources. All reactions were carried out under argon atmosphere using anhydrous solvents. Room temperature (rt) refers to 25 ± 2 °C. NMR spectra were recorded on a Bruker (Billerica, MA, USA) 400 MHz spectrometer. Chemical shifts are given in ppm (δ), calibrated to the residual solvent signals or TMS and internally calibrated by solvent frequency for ^19^F NMR. Exact mass measurements were performed on mass spectrometer (Q-TOF-2, Micromass-Waters, Milford, MA, USA) equipped with a standard electrospray ionization (ESI) and modular LockSpray TM interface. The RP-HPLC was performed using Phenomenex (Torrance, CA) Luna 5 µm C18(2)100A, AXIA, 21.2 × 250 mm column. Purity was determined using Agilent (Santa Clara, CA) C18-XDB, 5 µm, 4.6 × 250 mm column and a 0→100% linear gradient of acetonitrile/10 mM triethylammonium acetate as mobile phase. Purity of all the tested compounds was >95% at respective absorption wavelength in nm, unless noted otherwise.

*XAC245-AF488 (XAC488) triethylamine salt*. A stock solution of XAC245.TFA (Supporting information, 6.0 mg, 7.26 µmol, synthesized as reported [27]) and DIPEA (4.0 µL, 21.79 µmol) was made in anhydrous DMSO (596 µL). To a vial containing AlexaFluor488-5TFP ester (1.0 mg, 1.13 µmol), equipped with magnetic stir bar, was added XAC245 stock solution (150 µL, 1.70 µmol) under argon atmosphere. The reaction vial was covered with aluminum foil and the mixture stirred at rt for 18 h. RP-HPLC purification gave the product as an orange solid (0.88 mg, 59%, RP-HPLC linear gradient CH_3_CN-10 mM TEAA in H_2_O (*v*/*v*) 05/95→45/55 in 40 min, flow rate = 5.0 mL/min, R_t_ = 39.14 min). ^1^H-NMR (400 MHz, D_2_O) δ 8.20 (d, *J* = 1.9 Hz, 1H), 7.92 (dd, *J* = 7.9, 1.9 Hz, 1H), 7.72 (d, *J* = 8.5 Hz, 2H), 7.24 (d, *J* = 7.9 Hz, 1H), 6.98 (d, *J* = 9.3 Hz, 2H), 6.96–6.91 (m, 2H), 6.77 (d, *J* = 9.3 Hz, 2H), 4.53 (s, 2H), 3.98 (t, *J* = 7.5 Hz, 2H), 3.87 (t, *J* = 7.7 Hz, 2H), 3.47–3.33 (m, 5H), 3.29 (t, *J* = 6.8 Hz, 2H), 3.07 (t, *J* = 6.5 Hz, 2H), 2.32 (t, *J* = 6.8 Hz, 2H), 2.21 (t, *J* = 7.0 Hz, 2H), 2.05 (t, *J* = 7.2 Hz, 2H), 1.62 (dp, *J* = 15.8, 7.7 Hz, 8H), 1.51–1.33 (m, 7H), 0.89 (td, *J* = 7.4, 4.1 Hz, 6H). HRMS *m*/*z* [M − H]^−^ for C_56_H_65_O_17_N_11_S_2_ calculated 1226.3923, found 1226.3918.

*AF488-amide triethylamine salt*. To a solution of AlexaFluor-488-TFP ester (0.5 mg, 0.56 µmol) in anhydrous DMSO (150 µL) was added 2M NH_3_-iPrOH (6 µL, 11.3 µmol) and the mixture stirred in the dark at rt for 18 h. Purification by RP-HPLC afforded the product as an orange solid (0.78 mg, quantitative yield, RP-HPLC linear gradient, CH_3_CN-10 mM TEAA in H_2_O (*v*/*v*) 05/95→20/80 in 40 min, flow rate = 5.0 mL/min, R_t_ = 22.2 min). ^1^H NMR (400 MHz, D_2_O) δ 8.35 (d, *J* = 1.8 Hz, 1H), 8.07 (dd, *J* = 8.0, 1.9 Hz, 1H), 7.47 (d, *J* = 8.0 Hz, 1H), 7.24 (d, *J* = 9.3 Hz, 2H), 6.97 (d, *J* = 9.3 Hz, 2H). HRMS *m*/*z* [M + H]^+^ for C_21_H_15_O_10_N_3_S_2_ calculated 534.0277, found 534.0276.

*BY630-amide*. To a solution of BODIPY630/650-NHS ester (0.62 mg, 1.0 µmol) in anhydrous DMSO (150 µL) was added 2 M NH_3_-iPrOH (1.0 µL, 2.0 µmol) and the mixture stirred in dark at rt for 3 h. Purification by RP-HPLC afforded the product as blue solid (0.44 mg, 83%, RP-HPLC linear gradient, CH_3_CN-10 mM TEAA in H_2_O (*v*/*v*) 50/50→80/20 in 40 min, flow rate = 5.0 mL/min, R_t_ = 32.5 min). ^1^H-NMR (400 MHz, DMSO-*d*_6_) δ 8.14 (t, *J* = 5.9 Hz, 1H), 8.04 (dd, *J* = 3.8, 1.1 Hz, 1H), 7.84 (d, *J* = 5.1 Hz, 1H), 7.77 (s, 1H), 7.72 (s, 1H), 7.61 (dd, *J* = 6.2, 2.6 Hz, 3H), 7.44–7.34 (m, 2H), 7.34–7.26 (m, 3H), 7.22 (s, 1H), 7.12–7.04 (m, 2H), 6.95 (d, *J* = 4.2 Hz, 1H), 6.68 (s, 2H), 4.53 (s, 2H), 3.12 (q, *J* = 6.7 Hz, 3H), 2.02 (t, *J* = 7.4 Hz, 3H), 1.46 (dt, *J* = 15.5, 7.7 Hz, 6H), 1.25 (t, *J* = 7.8 Hz, 5H). ^19^F NMR (376 MHz, DMSO-*d*_6_) δ −137.81 (dd, *J* = 68.3, 33.3 Hz). HRMS *m*/*z* [M + Na]^+^ for C_29_H_29_O_3_N_4_F_2_B calculated 585.1919, found 585.1919. Purity was 98.1% at 630 nm.

## 3. Results

### 3.1. Functional Antagonism

To examine and analyze mechanistically the patterns of functional antagonism by various antagonists of different chemical entities, cells expressing relatively low A_2A_AR levels with minimal signaling amplification were used, as recommended [26], since receptor overexpression is related to the constitutive activity, and signaling amplification has been linked to changing the patterns of antagonism [28]. In this assay, the EC_50_ value of the agonist CGS21680 in stimulation of cAMP accumulation in HEK293 cells stably expressing the human A_2A_AR was measured to be ~15 nM, i.e., equivalent to the K_d_ value of [^3^H]CGS21680 determined by saturation binding (K_d_ = 15.5 ± 3.6 nM; B_max_ = 360 ± 48 fmol/mg protein).

**Figure 2:**
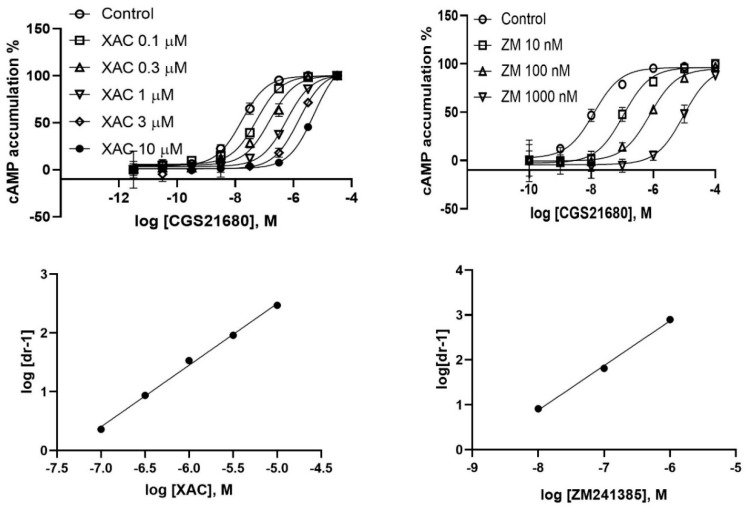
Antagonism of agonist CGS21680-induced cAMP production by XAC (upper left) and ZM241385 (upper right) in HEK293 cells expressing the human A_2A_AR. Cells were pretreated with antagonists for 20 min before agonist addition. Data are from three independent experiments. Lower left: Schild analysis of the antagonism by XAC. Lower right: Schild analysis of the antagonism by ZM241385.

In order to examine the pattern of antagonism by the putative bitopic ligands, we first examined the effects two known monotopic A_2A_AR antagonists, XAC and ZM241385, in antagonizing agonist CGS21680-induced cAMP accumulation on the agonist concentration-response curves, and Schild analysis was performed. Both XAC and ZM241385 shifted agonist concentration-response curves to the right in a concentration-dependent manner with slopes close to unity (Figure 2). The K_B_ and slope values calculated from the Schild analysis are listed Table 1. The binding of ZM241385 and XAC has been shown previously in the A_2A_AR crystal structures to occur in different poses. Dore et al. [29] showed that XAC binds in the same site as ZM241385, forming the same two key interactions. In comparison to the ZM241385-bound structure, XAC binding to the A_2A_AR induces a shift of Phe168 toward Val172 resulting in a displacement of the helical portion of extracellular loop 2 (EL2). However, XAC and ZM241385 did not show different binding patterns in the present study based on Schild analysis. This indicates that distinct binding poses of particular small molecule A_2A_AR ligands may not alter the competitive character of orthosteric binding. However, in other receptors a significant impact of the binding pose of noncanonical ligands on antagonist binding patterns has been demonstrated [30,31].

**Figure 3:**
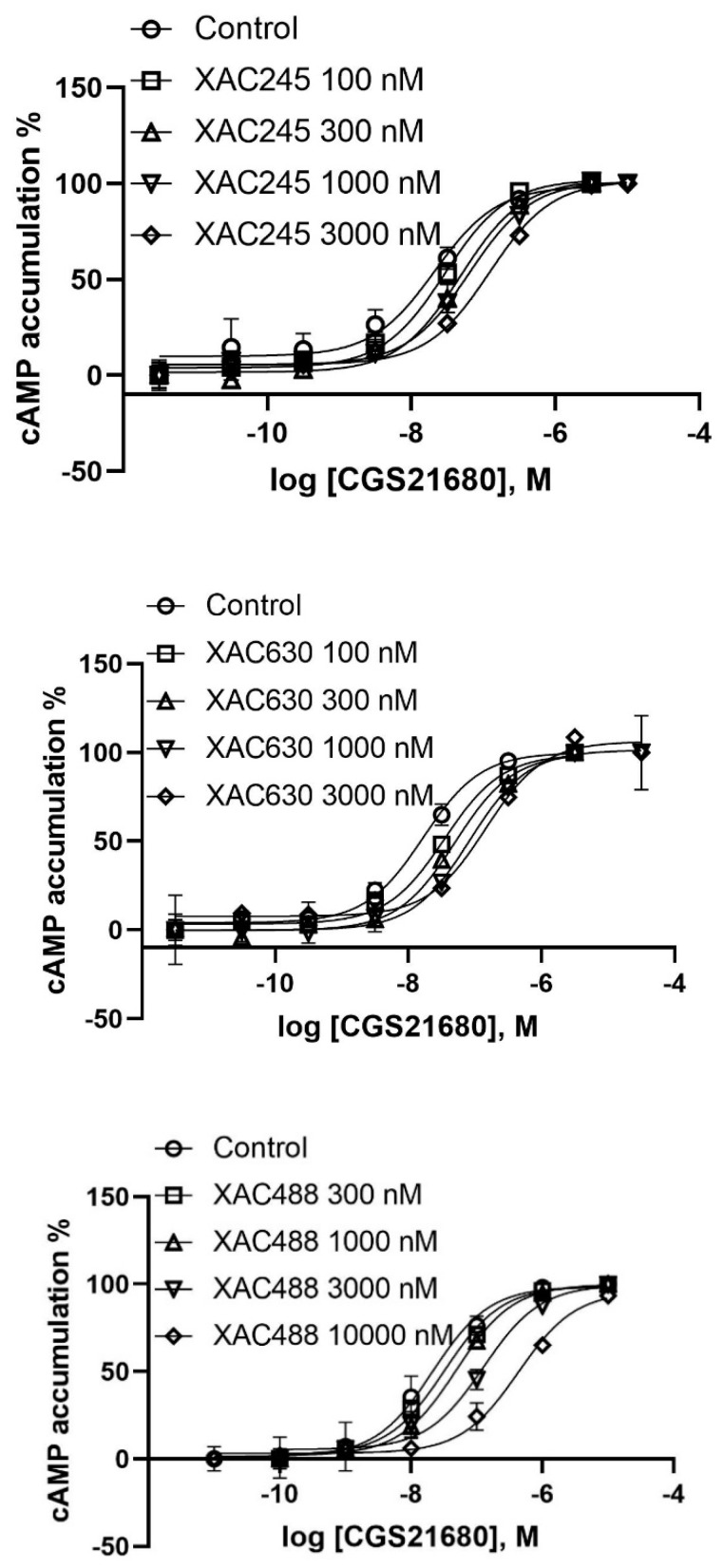
Effect of three XAC derivatives XAC245 (upper), XAC630 (middle) and XAC488 (lower) on the potency and efficacy of the A_2A_AR agonist, CGS21680, measured in a cyclic AMP accumulation assay using intact HEK293 cells stably expressing the human A_2A_AR. Agonist effect on the *y*-axis represents percent of cyclic AMP production. Data are representative of 2–4 separate experiments performed in duplicate or triplicate.

Next, we examined a known fluorescent derivative of XAC, XAC630, the binding property of which has been examined previously at the A_1_AR [15]. It has been shown that 1 µM XAC630 right-shifted NECA-induced inhibition of forskolin-stimulated cAMP accumulation in CHO cells expressing the human A_1_AR in a parallel manner [32]. XAC630 binding at the A_3_AR has also been investigated using a Nanobret binding assay [33]. It was found that the same unlabeled ligand showed different pharmacological parameters depending the labeled probe used (XAC630 vs. [^3^H]PSB-11) [33]. The property of A_2A_AR antagonism by XAC630 has not been previously characterized. Thus, in the present study we carefully examined its A_2A_AR binding characteristics and functional antagonism. The K_i_ value of XAC630 was calculated to be similar to that of XAC in displacing antagonist [^3^H]ZM241385 and agonist [^3^H]CGS21680 binding, but weaker in displacing the binding of AlexaFluor488 (AF488)-labeled tracer MRS7416 (Figure 3). Although XAC and XAC630 have similar K_i_ values in displacing radioligand binding, XAC630 right-shifted the concentration-response curve of CGS21680 with a pattern different from that of XAC (Figure 3). Schild analysis showed that XAC630 and XAC have similar K_B_ values (Table 1). However, the slopes of XAC and XAC630 are 1.06 ± 0.06 and 0.47 ± 0.04, respectively, which are significantly different (*p* < 0.01, student’s t-test). This result suggests that the conjugation of BY630 to XAC changed the antagonism pattern, which can be interpreted as allosteric rather than competitive antagonism. This finding prompted us to examine if the BY630 moiety alone has any effect on the A_2A_AR. Therefore, we synthesized as controls chemically stable amide derivatives of two fluorophores. However, it was found that neither BY630-amide nor AF488-amide alone (Figure 1C), at a concentration of 10 µM, had any A_2A_AR effect (Table 1). In addition to the large fluorophore, it is noted that the linker between XAC and BY630 moiety is also extended.

We next tested an extended amino congener of XAC with a similar but not identical alkylamide linker (XAC245, Figure 1C) to XAC630. Interestingly, extended amine congener XAC245 (2-4-5 representing the number of methylenes in the chain), originally synthesized in a study of A_3_AR fluorescent antagonists [27], was found to be weaker in displacing A_2A_AR radioligand binding compared to both XAC and XAC630. The K_B_ of XAC245 to shift the agonist response was roughly consistent with its binding affinity, although with a slope (0.68 ± 0.04) significantly different from both XAC and XAC630 (*p* < 0.05, one-way ANOVA). This result suggests that XAC245 is also endowed with an allosteric binding property. As AF488 is another commonly used fluorophore, the antagonism by AF488-conjugated XAC245 derivative (XAC488) was characterized. Interestingly, XAC488 was found to be weaker, in comparison with XAC and XAC630, in both binding and functional assays (Figure 3, Table 1). However, the slope (1.03 ± 0.05) of XAC488 from Schild analysis was found to be close to unity, consistent with a competitive binding mode. Thus, both the linker and fluorophore of may affect both potencies and patterns of antagonism of bitopic, fluorescent GPCR ligands.

Both the prototypical A_2A_AR antagonist SCH442416 and its aryl sulfonate derivative MRS7352 bind to A_2A_AR with roughly nM affinity [18]. The receptor interactions of two reported fluorescent antagonists, BY630-conjugated MRS7396 and AF488-conjugated MRS7416, which are based on functionalized congeners of SCH442416, were previously modeled computationally, but its functional antagonism has not been carefully characterized [18]. Briefly, the pyrazolotriazolopyrimidine core of fluorescent antagonist MRS7396 was predicted to establish the same interactions within the orthosteric binding site observed for the aryl sulfonate MRS7352. The hydrophobic Bodipy fluorophore and linker of MRS7396 pointed toward the extracellular side and folded back toward the TM bundles, driven its attraction to an aromatic pocket at the interface between TM1 and TM7. MRS7416 showed a unique binding mode featuring the more hydrophilic AF488 fluorophore stacked between EL2 and EL3 [18].

**Figure 4:**
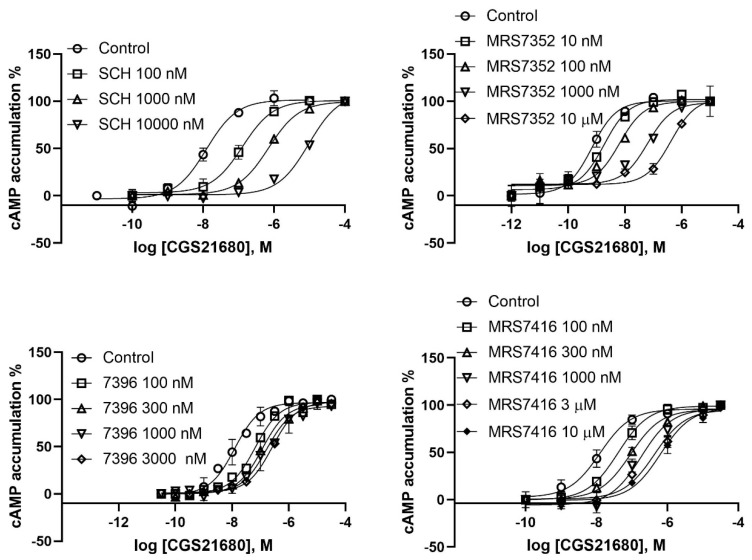
Effect of SCH442416 (upper left) and its three derivatives MRS7352 (upper right), MRS7396 (lower left) and MRS7416 (lower right) on CGS21680-induced cyclic AMP accumulation assay in intact HEK293 cells stably expressing the human A_2A_AR. Data are from three independent experiments performed in duplicate or triplicate.

**Figure 5:**
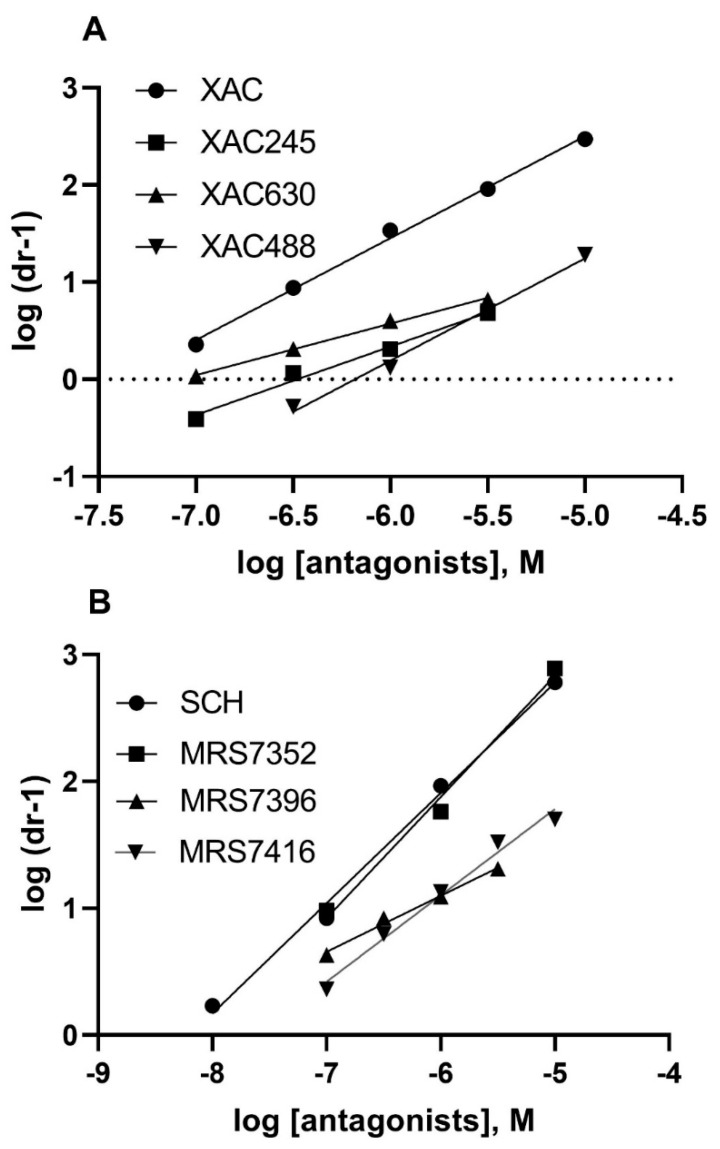
Schild analysis of the antagonism at the A_2A_AR by XAC derivatives (**A**) and SCH442416 derivatives (**B**). The K_B_ and slopes values are summarized in Table 1. It is notable that at a concentration of 10 µM, XAC630 or MRS7396 did not produce further rightward shift of the agonist concentration-response curve. Thus, for Schild analysis, only the linear part of the plot was used.

In the present study, we further analyzed functional antagonism patterns at the A_2A_AR of this antagonist family. The K_B_ values of both SCH442416 and MRS7352 were similar to their K_i_ values obtained from radioligand binding, with slopes close to 1 (Figure 4; Table 1). The K_B_ values of MRS7396 and MRS7416 were 1.70 ± 0.34 and 9.12 ± 1.77 nM, respectively. However, the slopes of MRS7396 and MRS 7416 were 0.42 ± 0.02 and 0.68 ± 0.03, respectively (Figure 5; Table 1). Thus, different patterns of antagonism were observed for conjugates MRS7396 and MRS7416 in comparison to their parent compound SCH442416 and related sulfonate MRS7352.

**Figure 6:**
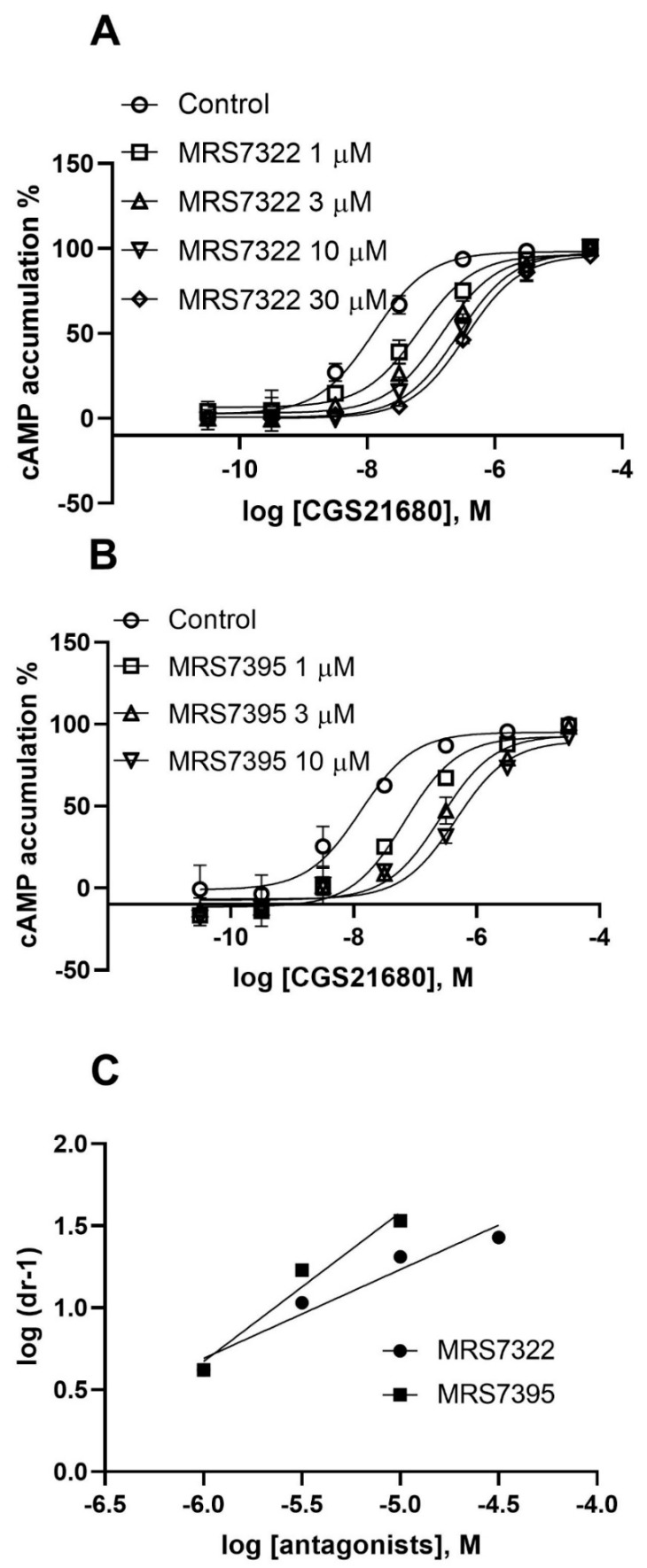
Antagonism by MRS7322 (**A**) and MRS7395 (**B**) and Schild analysis of the antagonism (**C**). The K_B_ and slopes values are summarized in Table 1. Data are from three independent experiments performed in triplicate.

Two weaker fluorescent antagonists MRS7322 (compound **9** in Duroux et al. [18], with a two-methylene spacer) and MRS7395 (compound **10** in Duroux et al. [18], with a four-methylene spacer) are both AlexaFluor647-conjugated ligands based on SCH442416 with the only difference being linker length. It was indicated based on functional antagonism and Schild analysis that MRS7322 and MRS7395 are allosteric and competitive antagonists, respectively (Figure 6; Table 1), which is consistent with an earlier finding from Baker et al. [15] that XAC fluorescent derivatives with different linker lengths showed distinct A_1_AR binding properties.

**Figure 7:**
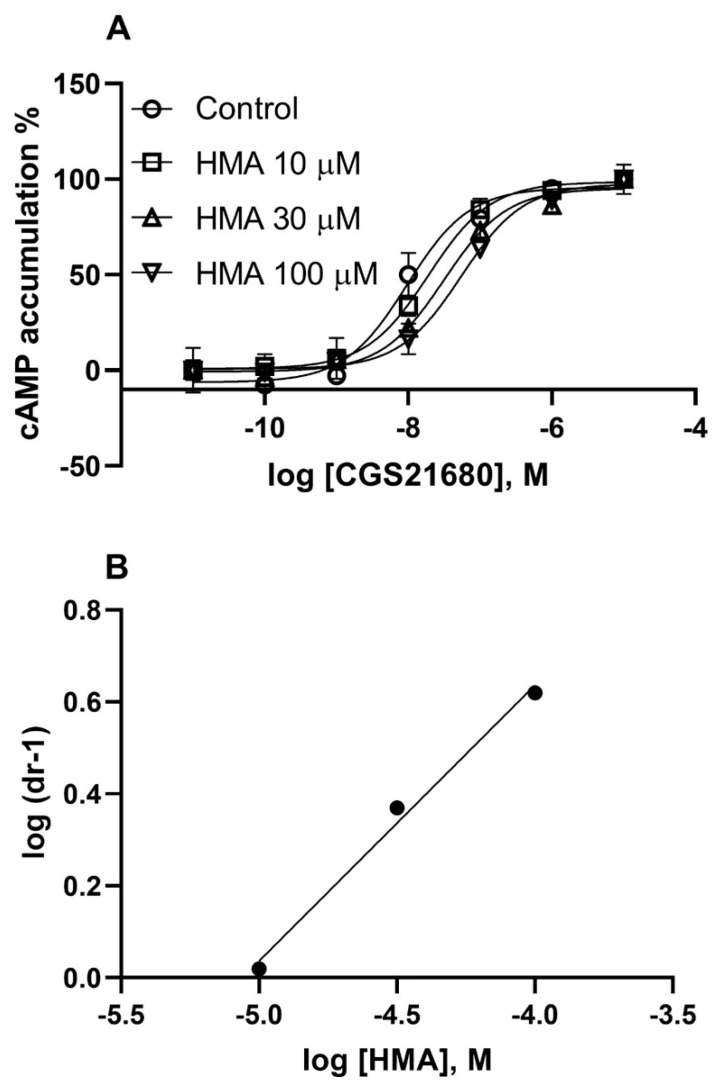
Functional antagonism of CGS21680-induced cyclic AMP accumulation in HEK293 cells expressing the A_2A_AR. Agonist effect on the *y*-axis represents percent of cyclic AMP production. Cells were pretreated with HMA 20 min before the addition of agonist CGS21680. (**A**). Shift of CGS21680 concentration-response curve by HMA. (**B**). Schild analysis. Results are from three separate experiments performed in duplicate. The KB and slopes values are summarized in Table 1.

In addition to comparing the bitopic ligands with their parent competitive antagonist molecules, SCH442416 and XAC, a known allosteric modulator, 5-(*N*,*N*-hexamethylene)amiloride (HMA), was also characterized. HMA has been reported as an allosteric A_2A_AR modulator [4,10,11]. However, to our knowledge, the nature of functional antagonism by HMA at the A_2A_AR has not been determined. Thus, in order to compare the patterns of allosteric antagonism by HMA and bitopic ligands, we examined the ability of HMA to shift the concentration-response curve of the agonist CGS21680-induced cAMP accumulation in HEK293 cells expressing the A_2A_AR (Figure 7). HMA showed a K_B_ value of 3810 ± 450 nM, roughly consistent with its K_i_ value obtained from displacing [^3^H]ZM241385 binding (3050 ± 1210 nM). A slope value of 0.59 ± 0.05 was calculated from Schild analysis, consistent its allosteric binding property. It is noted that, at a concentration of 0.3 mM, HMA did not significantly shift the agonist concentration-response curve further to the right. Thus, for Schild analysis, only the linear part of the plot was used.

### 3.2. Binding

**Figure 8:**
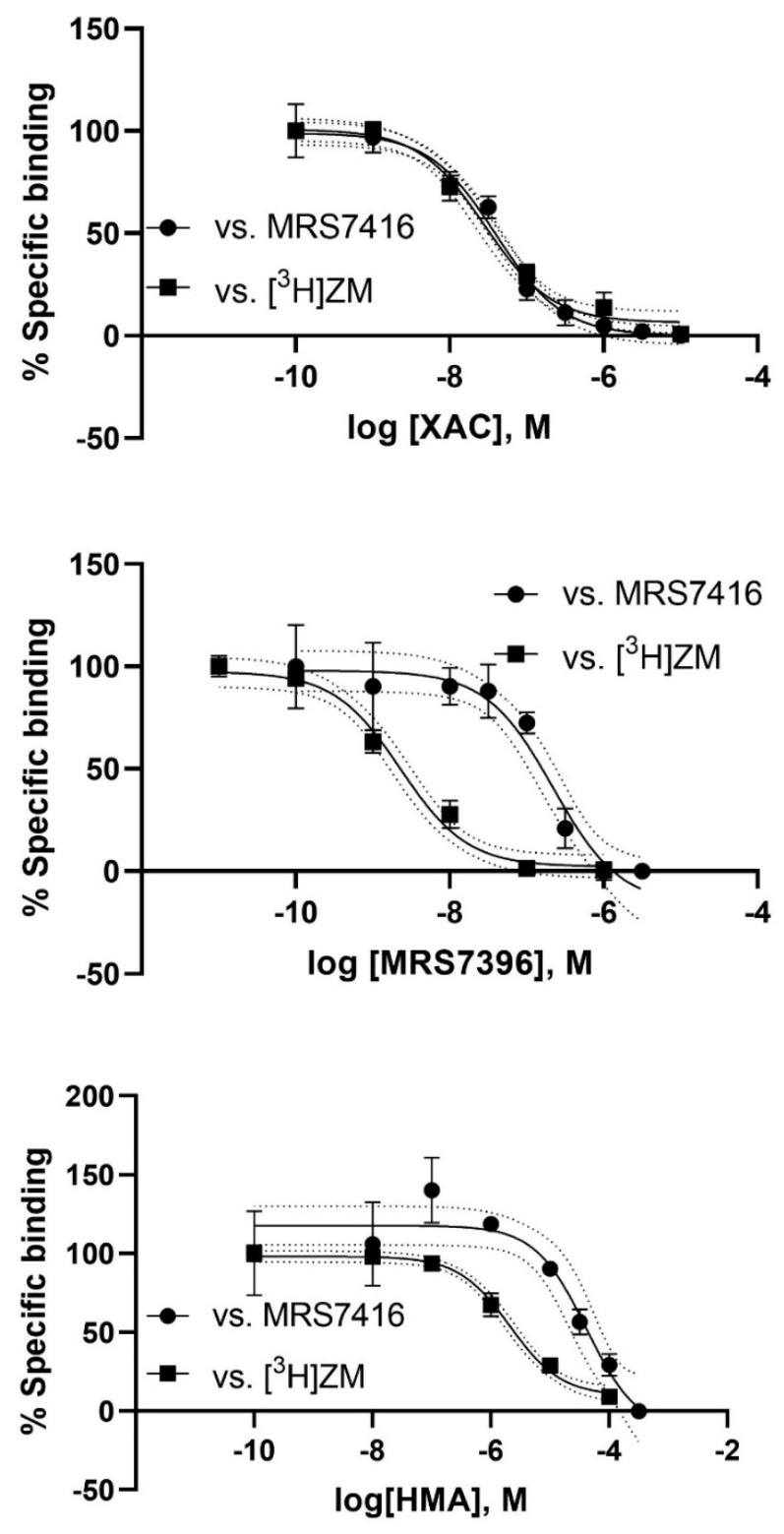
Effects of the selection of labeled ligands on the determination of K_i_ values of unlabeled ligands from different chemical classes. Upper: XAC; middle: MRS7396; lower: HMA. Cell membrane preparations were used in [^3^H]ZM241385 ([^3^H]ZM) binding. MRS7416 binding was measured using intact cells. Data were from three separate experiments providing similar results performed in duplicate.

In addition to the analysis of the patterns of functional antagonism, we also compared the ability of various agonists, antagonists and especially bitopic ligands to displace the binding of agonist radioligand [^3^H]CGS21680, antagonist radioligand [^3^H]ZM241385 and the AF488-conjugated fluorescent ligand MRS7416. HEK293 cells expressing the A_2A_AR were used in all these binding experiments. Of the antagonists tested, XAC, XAC245, XAC488, SCH442416, MRS7352 showed binding K_i_ values similar to K_B_ values from functional antagonism (Table 1). Interestingly, MRS7396, XAC630 and HMA were 9–100 times weaker in displacing MRS7416 binding than radioligand binding (Figure 8). Of the agonists tested, CGS21680 and NECA were weaker in displacing MRS7416 binding than in displacing [^3^H]ZM241385 and [^3^H]CGS21680 binding. However, adenosine agonist UK432097 and 3,5-pyridinecarbonitrile agonist LUF5834 had similar K_i_ values in displacing MRS7416 binding and radioligand binding (Table 1).

**Figure 9:**
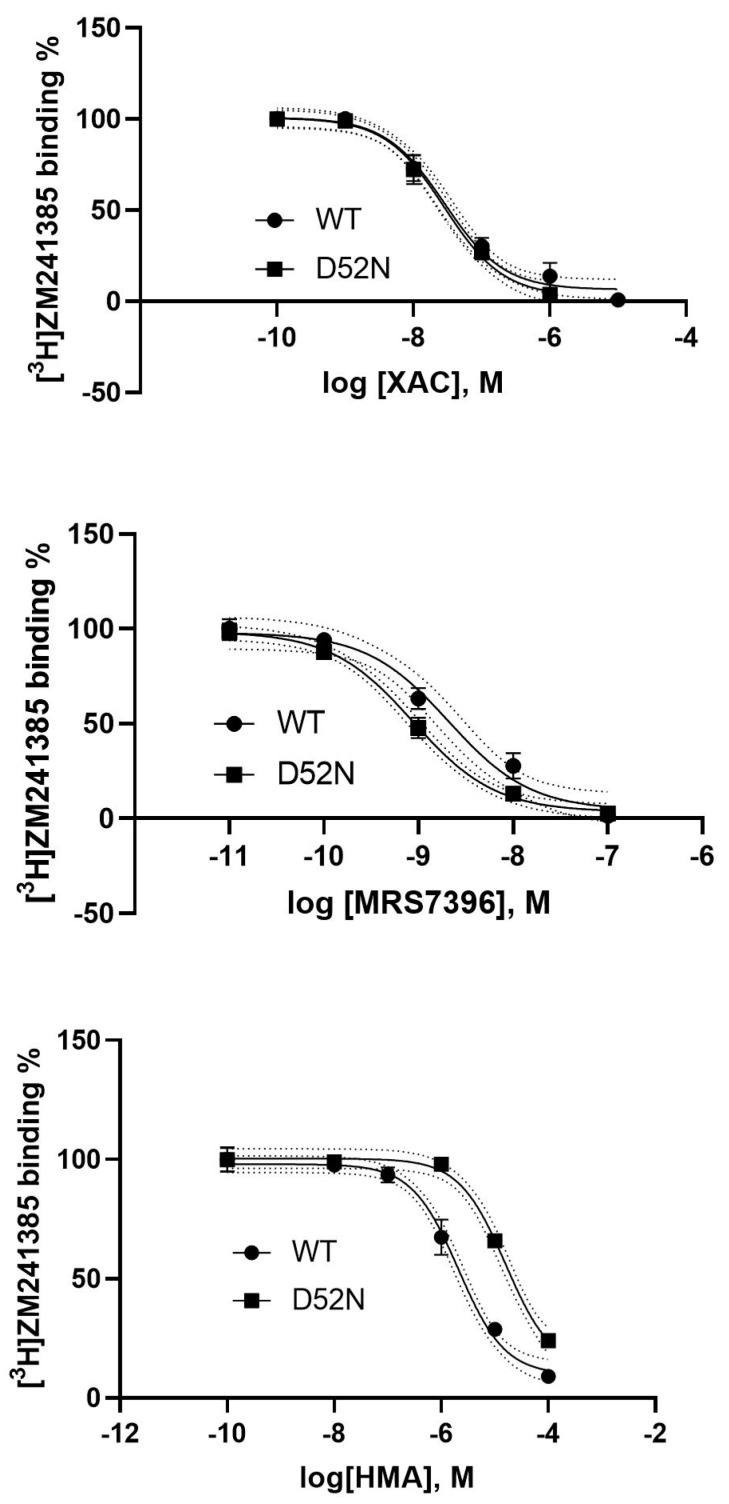
Distinct effect of the sodium mutation on the binding of XAC (upper), MRS7396 (middle) and HMA (lower). Data were from three independent experiments performed in duplicate. The antagonist radioligand [^3^H]ZM241385 (1 nM) was used in this experiment.

It is known that mutation of Asp52 (D) at the A_2A_AR sodium site impaired its activation and changed it to an inactive state [6,8,10]. Here we examined if HMA and MRS7396 behave in a similar manner or not. Figure 9 shows that XAC had similar affinity for WT (32.1 ± 5.9 nM) and D52N (31.9 ± 5.6 nM) mutant A_2A_ARs expressed in HEK293 cells. HMA showed a 5-fold affinity decrease at D52N. The affinities of HMA in WT and D52N were 3050 ± 1210 and 15,800 ± 2160 nM, respectively. The K_i_ of MRS7396 was 2.34 ± 0.33 nM at WT, i.e., ~3-fold weaker than at the D52N mutant receptor (0.79 ± 0.23 nM). Thus, it is possible that MRS7396 and HMA prefer different A_2A_AR conformational states.

### 3.3. Mathematical Modeling

The experimental curves representing the effects of an A_3_AR allosteric enhancer on the efficacy of various agonists in stimulating various signaling pathways have been simulated previously using theoretical parameters [34]. In the present study, we extended this mathematical modeling by simulating the effects of allosteric antagonists on the stimulation of A_2A_AR-mediated cAMP accumulation by agonist CGS21680. The equations from Hall [26] and experimental curves from the measurement of cAMP accumulation in the presence or absence of allosteric antagonists were used as a basis for simulation of experimental curves and to derive conditions for variation of binding cooperativity (*γ*) and association constant (*M*) of the allosteric modulators. The fraction of active state receptors within the total receptor pool can be expressed as: [*R*_active_]/[*R*]_T_ = ([R*] + [AR*] + [R*B] + [AR*B])/*RT*, which can be re-stated as: [*R*_active_][*RT*] = *L*(1 + *αK*[*A*] + *βM*[*B*](1 + *αγδK*[*A*]))1 + *L* + *M*[*B*](1 + *βL*) + *K*[*A*](1 + *αL* + *γM*[*B*](1 + *αβδL*)), where *L* is the receptor isomerization constant (the ratio of active state receptor over the inactive state), *K* is the equilibrium association constant of the orthosteric ligand *A*, *M* is the equilibrium association constant of ligand *B* (allosteric modulator), *α* is the intrinsic efficacy of ligand *A*, *β* is the intrinsic efficacy of ligand *B*, *γ* is the binding cooperativity between *A* and *B*, *δ* is the activation cooperativity between *A* and *B* and *L* is only related to the receptor.

**Figure 10:**
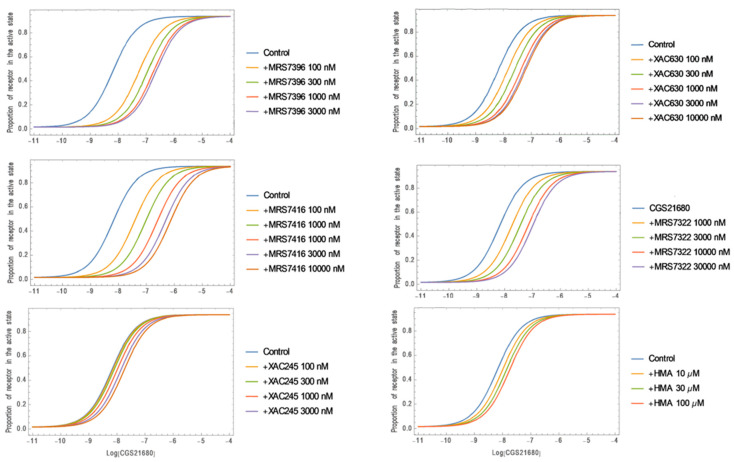
Simulation of concentration-response curves with Wolfram Mathematica software. The equations from Hall [26] were used to derive conditions that vary patterns and potencies. Parameters in these equations are listed in the text.

We simulated the concentration–response curves for CGS21680 in the absence and presence of various concentrations of allosteric antagonists (parameter settings in Table 2). In all instances, we assumed an identical fraction of active receptors (*L*), identical equilibrium dissociation constants for both orthosteric (*K*) and identical intrinsic activity of the allosteric modulator (*β* = 1, since the allosteric modulators alone do not activate the receptor) and identical activation cooperativity (*δ* = 1, as the allosteric antagonists do not affect agonist E_max_). The experimental curves of CGS21680 in the absence and presence of allosteric modulators were closely simulated by varying only the association constant values of allosteric modulator (*M*) and the binding cooperativity between orthosteric and allosteric ligands (*γ*) (Figure 10).

The simulation results may explain mechanistically the different patterns and antagonism potencies of MRS7396 and MRS7416, as probably due to both their different binding cooperativities with the orthosteric site and their different association binding constants. It seems that the extent of rightward shift is mainly due to binding cooperativity, since a very high negative cooperativity is required to simulate the antagonism by MRS7416. However, for MRS7396 a high association binding constant as an allosteric modulator is needed for potent inhibition of receptor binding, since MRS7396 is 10-fold more potent in binding but less potent functionally, in comparison to MRS7416. Considering the antagonism by both MRS7396 and MRS7416, it seems that the Schild K_B_ values are consistent with their binding K_i_ values, which is due to their slope differences, but inconsistent with the extent of rightward shift of concentration-effect response curves. Strikingly, MRS7416 produced a larger shift than MRS7396 at 3 µM, although MRS7416 was weaker in displacing radioligand binding.

Two Bodipy-630 conjugated derivatives, XAC630 and MRS7396, showed similar patterns of antagonism with a slope < 0.5. The reason that MRS7396 is more potent than XAC630 functionally is related to both a more negative binding cooperativity and a larger association constant of MRS7396. The possible explanation that MRS7416 is more potent than XAC245, MRS7322 and HMA seems only partly due to a larger association binding association constant, but probably mainly due to the very negative binding cooperativity of MRS7416. Based on the previous crystal structures and modeling, apparently HMA and the bitopic ligands act at different allosteric sites on the A_2A_AR.

## 4. Discussion

Hybrid molecules that concomitantly engage both an orthosteric and an allosteric site on a receptor have been termed “bitopic”, “dualsteric” or “bivalent” ligands [19,21,23,24,25,35,36]. As such, bitopic ligands are a distinct ligand class with respect to monovalent ligands, which target only a single receptor site. The development of bitopic ligands has proven attractive, because bivalent ligands bear two target-binding pharmacophores that bind to two distinct binding sites at the target and permit the distinct recognition moieties to exert allosteric modulation of each other’s affinity via cooperativity. Consistent with this concept that two distinct chemical moieties in one ligand molecule can bind two different receptor domains [17], results from modeling, mutagenesis, pharmacological characterization, especially recent crystal structures of various GPCRs, indeed demonstrated that two molecules can bind individually or simultaneously to distinct receptor sites [30,37,38]. Thus, each receptor may have one orthosteric site and multiple allosteric sites, and bitopic ligands may bind to the same orthosteric site but different allosteric sites depending on the poses, linkers and other structural features [18]. Heitman et al. [39] have shown that the amiloride analog HMA and another antagonist bind with neutral cooperativity to two distinct allosteric sites in the gonadotropin-releasing hormone receptor.

Bitopic fluorescent ligands have been reported for all four AR subtypes [13,14,15,16]. Baker et al. [15] studied the effects of A_1_AR bitopic ligands and concluded that the pharmacology of a fluorescent ligand was critically influenced by both the fluorophore and the associated linker. It has been suggested that unlike the tritium labeling of AR ligands, the pharmacology of a small molecule antagonist changes considerably with a conjugated fluorophore [15,32]. The nature of A_2A_AR antagonism by fluorescent antagonists, including XAC630, at the A_2A_AR has not been extensively characterized and analyzed mechanistically, although several have been shown to be A_2A_AR antagonists [16,18] suitable as receptor binding tracers. More recently, various fluorophores have been conjugated to A_2A_AR antagonist preladenant and have been modeled and pharmacologically characterized using a bioluminescence resonance energy transfer (NanoBRET) assay [40].

Ligand binding is a complex process, e.g., more than one subunit of nicotinic acetylcholine and GABA_A_ receptors is involved in agonist binding and inter-subunit cooperativity exists. Kirtley and Koshland [41] have modeled ligand interactions with multi-subunit proteins. For monomeric rhodopsin-like GPCRs, ligands can induce conformational changes within the same protein, which is a different kind of cooperativity. It has been suggested that the classical theory such as Schild analysis may not apply to ligand having two non-equivalent binding sites [42,43]. However, based on present results, it seems that Schild analysis can remain accurate for bitopic antagonists (e.g., XAC-BY630, MRS7396 and MRS7416) even though the slopes significantly deviated from unity, i.e., the Schild K_B_ values are roughly consistent with K_i_ values from radioligand binding. This further highlighted the usefulness of Schild analysis [42,44]. Kenakin [45] has suggested that functional experiments can provide more accurate estimates of functional affinity, which are more relevant to net receptor activity, and suggested not utilizing binding affinities as functional descriptors. Thus, binding experiments may be misleading depending on the labeled ligands used.

The functional characterization of other allosteric antagonists has been well documented [46]. Allosteric antagonists that do not depress the maximal response yield dose-ratios for an orthosteric agonist similar to those produced by competitive antagonists. In Schild analysis, competitive antagonists produce dose-ratios for the agonist that display a linear relationship [47]. Orthosteric antagonists sterically hinder orthosteric agonist binding, which becomes unsaturable, and a Schild regression is purely linear. However, allosteric antagonists bind to a different receptor site, and the antagonism of orthosteric agonists becomes saturable when the allosteric site is fully occupied. Thus, at higher concentrations, allosteric antagonists cease to further right-shift the agonist concentration-response curve, and the dose-ratio reaches a maximal value, and therefore the Schild regression becomes nonlinear. However, when the linear portion of the Schild plot is subject to analysis, the slopes are often changed. It is interesting to note from the present study that the K_B_ values obtained from the Schild plot linear portions correlated with K_i_ values from displacement of antagonist [^3^H]ZM241385 binding, but not with displacement of the bitopic fluorescent antagonist MRS7416, suggesting a unique pattern of allosteric antagonism by bitopic ligands and the usefulness of Schild analysis.

The more potent amiloride analog, 3-amino-6-chloro-5-(1-homopiperidyl)-*N*-(diaminomethylene)pyrazinecarboxamide (HMA), which was proposed to bind to the sodium site [10,11], has been demonstrated to enhance the receptor population of active states but distinct from that activated by full agonists NECA and UK432097, and the inactive state stabilized by antagonist ZM241385 [48]. The present results are consistent with those earlier studies of HMA and the antagonist XAC showing different binding patterns. It is also interesting to note that D52N did not affect orthosteric agonist or antagonist binding, but decreased HMA binding affinity and increased MRS7396 binding affinity, respectively, which indicate that MRS7396, XAC and HMA may prefer binding to different A_2A_AR conformations. HMA has been shown to enhance the dissociation of antagonist [^3^H]ZM241385 but not agonist [^3^H]CGS21680 [4,49]. We demonstrate here that both HMA and MRS7396 displace the binding of [^3^H]ZM241385 and MRS7416 but with different binding affinities, although XAC showed same binding affinities in displacing the binding of MRS7416 and [^3^H]ZM241385, further suggesting potentially different binding modes of XAC, HMA, MRS7396 and MRS7416.

Ehlert and Griffin [50] modeled and analyzed the allosteric effect of gallamine at the M_2_ muscarinic receptor. By exhibiting negative cooperativity, gallamine only affects the agonist affinity but not efficacy, which is similar to HMA and MRS7396 effects at A_2A_AR, although HMA and MRS7396 may bind with different receptor conformational states or shift receptor conformational populations [48]. In contrast, the A_3_AR allosteric enhancer LUF6000 only affects agonist efficacy, but not binding affinity due to its activation cooperativity with orthosteric agonists [34].

Massink et al. [10,11] showed that the D52A mutation decreased HMA affinity by over 10-fold. The finding that D52A cannot be activated suggests it enforces an inactive conformation. A_2A_AR binding modes of amiloride and its bulkier analog HMA were determined by flexible side-chain docking where the charged guanidinium group of amiloride interacted with Asp52^2.50^ [5]. Gutierrez-de-Teran et al. [9] suggested that amiloride analogs can also bind to the sodium binding pocket, showing distinct patterns of agonist and antagonist modulation. Sodium slightly increases antagonist binding and decreases agonist binding, while HMA increased B_max_ of agonist [^3^H]NECA binding and decreased B_max_ of antagonist [^3^H]ZM241385 binding. HMA was more potent in displacing agonist [^3^H]NECA binding and antagonist [^3^H]ZM241385 binding, consistent with HMA promoting a specific A_2A_AR active state [48]. White et al. [8] found that D52N cannot be activated, and D52N has decreased affinity for HMA, also suggesting that HMA favors an active conformation, rather than or in addition to its possible interaction with the sodium site. Multiple conformational states have also been reported in many GPCRs, e.g., the serotonin 5-HT_2A_R [51] and A_2A_AR [48,52,53]. It is suggested that ligands of different classes could label receptors of different conformations.

It should be noted that radioligand and fluorescent ligand binding experiments have been done under different conditions. Radioligand binding to membrane preparations was undertaken at 25 °C in 50 mM Tris-HCl buffer, while fluorescent ligand binding was performed in intact cells incubated at 37 °C in normal growth media. The whole cell binding assay required cell detachment with EDTA, which may contribute to ligand dissociation. Thus, different assay conditions might contribute to the observed difference in the binding affinity of XAC630 from two different assays. However, the monotopic ligands, such as XAC and SCH442416, showed similar binding affinities in these two different binding assays. It is also notable that both XAC245 and XAC630 induced relatively smaller rightward shifts of the agonist concentration-response curve in comparison with XAC, although XAC630 and XAC have similar binding affinity in displacing radioligand binding. Thus, unlike the Schild analysis of the antagonism by MRS7396, MRS7416 and XAC with a wider range of shifts, the narrow range of shifts by XAC630 and XAC245 could somewhat introduce errors in the calculation of slope and K_B_ values. Nevertheless, the limited extent of shifts allowed a Schild analysis and produced a linear part in the Schild plot although with a lower slope. Despite the small shifts, the obtained K_B_ values were consistent with their K_i_ values obtained from radioligand binding experiments. It is also notable that there could be differences in ligand-binding kinetics for both labeled and unlabeled ligands, which should be taken into account in data interpretation. This is particularly pertinent for the FACS analysis in intact cells, where the washout period and EDTA incubation to disrupt cell monolayers might influence the binding constants obtained. Thus, ligands with different kinetic properties may be affected differently.

Although the patterns of A_2A_AR allosteric antagonism by bitopic ligands have not yet been extensively examined, allosteric modulation by bitopic ligands has been well-characterized in other GPCRs. As a portion of each bitopic ligand typically points toward a receptor distal region, these ligand extensions have been suggested to bind to various allosteric sites to produce a cooperative effect on overall receptor binding affinity of the ligand. Gaiser et al. [23] explored the bitopic ligand binding to both the orthosteric and metastable (allosteric) sites of the β_2_ adrenergic receptor and suggested that the linkers alone decreased the affinity at the orthosteric site, with hydrophobic receptor interactions of the allosteric portion. Tahtaoui et al. [19] reported fluorescent pirenzepine derivatives as potential human M_1_ muscarinic receptor bitopic ligands. Binding of Bodipy derivatives (linkers of 10–12 atom lengths) was sensitive to the presence of the allosteric modulator brucine, while that of all other molecules (linkers of 15–24 atom lengths) was not. The authors suggest that those analogues interact with both the orthosteric and allosteric sites. Valant et al. [54] found a hybrid molecule that produced biased A_1_AR signaling. The authors suggest that the concomitant association with both orthosteric and allosteric sites is the basis to display biased agonism. Two groups of Bodipy pirenzepine derivatives exhibited distinct allosteric binding poses [36]. The linker may dictate pharmacological outcomes for bitopic molecules that are hardly predictable from the properties of individual orthosteric and allosteric building blocks. The authors demonstrated that the fusion of a fluorophore to an orthosteric ligand is not neutral, as it may confer unexpected properties to the resultant fluorescent tracer. The mixed orthosteric/allosteric behavior is observed for many bitopic ligands [20,24,54]. Campbell et al. [25] reported that the conjugation of two 9-aminoacridine pharmacophores, using linkers of varying length, increases the allosteric potency and efficacy of this ligand at α_1_-adrenergic receptors, likely through optimization of bitopic engagement of the allosteric and orthosteric binding sites. Kumar et al. [21] synthesized and pharmacological characterization of novel *trans*-cyclopropylmethyl-linked bivalent ligands with dopamine D_3_ receptor (D_3_R) allosteric interactions. Fasciani et al. [55] suggested that SB269,652 behaves as a bitopic antagonist at unoccupied dopamine D_3_R, binding simultaneously to both orthosteric and allosteric sites, and as a pure negative allosteric modulator when receptors are occupied, and it can solely bind to an allosteric site. Notably, all bitopic ligands are allosteric, for example, MRS7395 and XAC488 are demonstrated to be competitive antagonists in nature.

In summary, the present study demonstrated that bitopic ligands could be potent, weak, competitive or allosteric based on the combination of the A_2A_AR antagonist pharmacophore, linker and fluorescent moieties. The binding affinity (K_i_) or functional potency (K_B_) of the bitopic antagonists is often related their negative binding cooperativity and other allosteric properties, but may not be related to the extent of rightward shift of agonist concentration-response curves. Thus, XAC630 and MRS7396 have higher binding affinity than XAC488 and MRS7416, respectively, but smaller rightward shift of agonist concentration-response curves in function. Therefore, bitopic ligands that are potent in binding may or may not be potent functionally. This could also be true for allosteric modulators other than bitopic ligands, which is in line with the saturable property of allosteric modulation. The potency of unlabeled ligands might be dependent on the labeled ligands used. Bitopic A_2A_AR antagonism could produce a unique pattern of antagonism, but it remains to be explored if this could be a therapeutic advantage or not.

## Figures and Tables

**Table 1:** Binding affinity and functional potency of various antagonists determined in radioligand binding, fluorescent ligand binding and a cyclic AMP functional assay using HEK293 cells stably expressing the human A_2A_AR. Refer to Figure 1 for structures.

		Binding (K_i_, nM)		Functional Antagonism	
	MRS7416	[^3^H]ZM241385	[^3^H]CGS21680	K_B_ (nM)	Slope
XAC	50.7 ± 9.3	32.1 ± 5.9	38.3 ± 6.7	46.6 ± 12.3	1.06 ± 0.06
XAC245	152 ± 29	198 ± 33	220 ± 36	280 ± 33	0.68 ± 0.04
XAC630	692 ± 173	36.6 ± 10.1	29.0 ± 5.7	51.1 ± 12.2	0.47 ± 0.04
XAC488	NA	388 ± 62	438 ± 110	650 ± 120	1.03 ± 0.05
SCH442416	7.31 ± 2.22	4.35 ± 0.89	5.01 ± 0.44	7.43 ± 1.85	1.05 ± 0.04
MRS7352	5.28 ± 1.31	6.24 ± 2.42	4.98 ± 1.13	6.61 ± 0.88	0.88 ± 0.06
MRS7396	256 ± 33	2.34 ± 0.33	2.83 ± 0.20	1.70 ± 0.34	0.42 ± 0.02
MRS7416	17.6 ± 4.1 ^a^	23.8 ± 5.6	16.4 ± 1.6	9.11 ± 1.77	0.68 ± 0.03
MRS7322	ND	108 ± 33	ND	52.5 ± 13.7	0.54 ± 0.09
MRS7395	ND	295 ± 176 ^c^	ND	182 ± 38.6	0.91 ± 0.07
ZM241385	1.83 ± 0.47	1.57 ± 0.32 ^b^	1.86 ± 0.53	1.35 ± 0.41	0.995 ± 0.055
HMA	18500 ± 2800	3050 ± 1210	2120 ± 510	3810 ± 450	0.59 ± 0.05
AF488-amide	ND	ND	3.7 ± 1.2%	NA	NA
BY630-amide	ND	ND	2.6 ± 1.5%	NA	NA
CGS21680	224 ± 23	68.6 ± 13.1	16.6 ± 3.2	NA	NA
NECA	377 ± 128	58.8 ± 12.2	23.7 ± 5.5	NA	NA
UK432097	23.3 ± 3.9	ND	12.1 ± 4.0	NA	NA
LUF5834	23.0 ± 4.6	13.3 ± 6.5	ND	NA	NA

Results are expressed as mean ± SEM from at least 3 independent experiments. ND, not determined; NA, not applicable. ^a^ K_d_ value from MRS7416 saturation experiments (*n* = 6); ^b^ K_d_ value from [^3^H]ZM241385 saturation binding. ^c^ Duroux et al. [18].

**Table 2:** Parameters used in Wolfram Mathematica simulations.

Antagonist	*L*	*K*	*M*	*A*	*β*	*γ*	*δ*	[*R*]
MRS7396	0.015	1 × 10^7^	1 × 10^8^	1000	1	0.025	1	1
MRS7416	0.015	1 × 10^7^	2 × 10^7^	1000	1	0.007	1	1
XAC245	0.015	1 × 10^7^	1 × 10^6^	1000	1	0.3	1	1
XAC630	0.015	1 × 10^7^	1.5 × 10^7^	1000	1	0.1	1	1
HMA	0.015	1 × 10^7^	1 × 10^5^	1000	1	0.3	1	1
MRS7322	0.015	1 × 10^7^	2 × 10^6^	1000	1	0.03	1	1

## Supplementary Materials

The following are available online at https://www.mdpi.com/2073-4409/9/5/1200/s1, Synthesis of XAC245.
