# Supplementary material for: Allosteric Antagonism of the A2A Adenosine Receptor by a Series of Bitopic Ligands
Source: Cells. 2020 May 12;9(5):1200. doi: 10.3390/cells9051200 (PMC7290864; doi:10.3390/cells9051200)
Supplement: Supplementary file 1 [file cells-09-01200-s001.pdf]

Supporting information

Allosteric antagonism of the A2A adenosine receptor by a series of bitopic ligands

Zhan-Guo Gao, Kiran Toti, Ryan Campbell, R. Rama Suresh, Huijun Yang and Kenneth A. Jacobson

Synthesis of XAC-245.TFA:

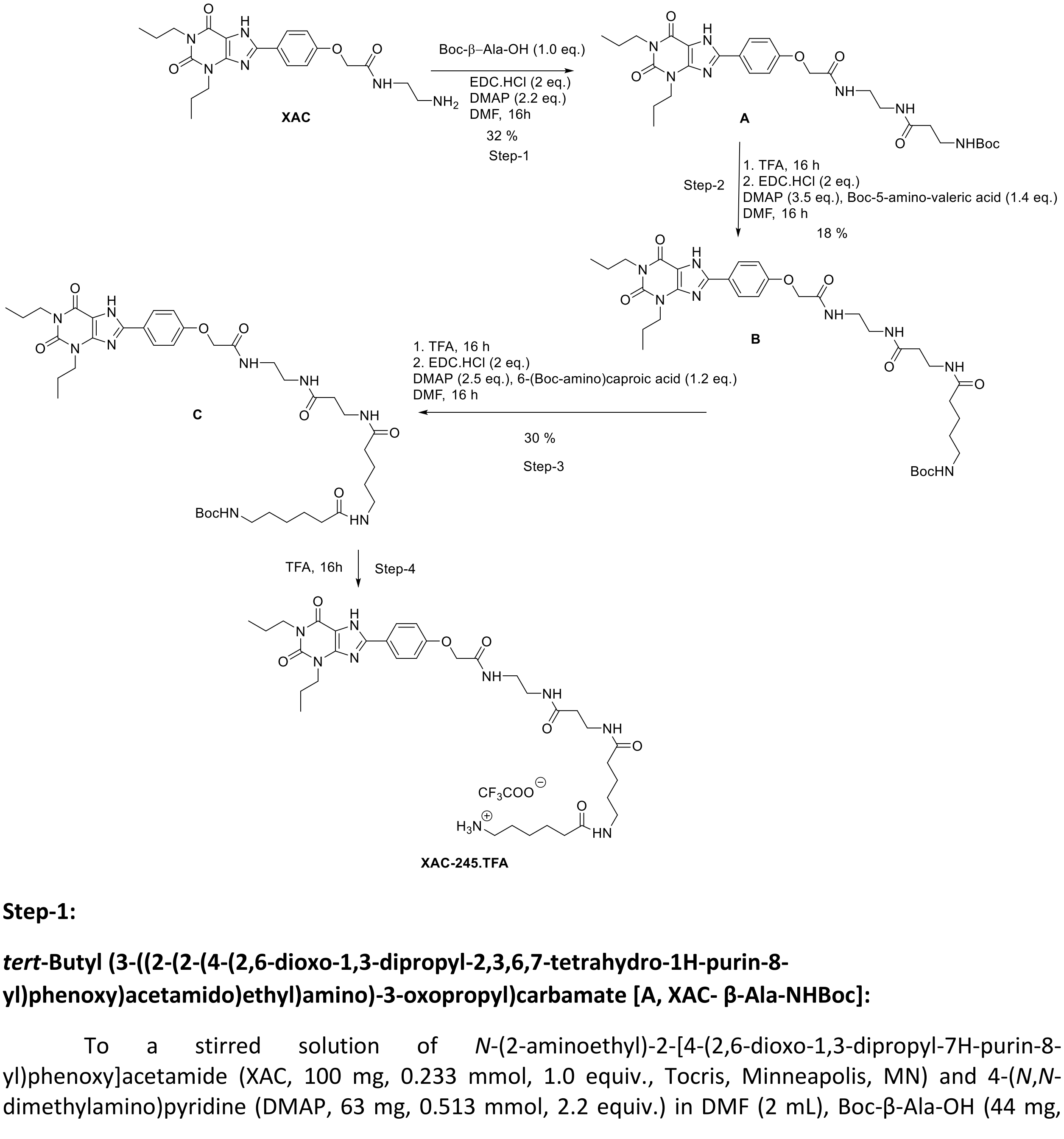

Step-1:

tert-Butyl (3-((2-(2-(4-(2,6-dioxo-1,3-dipropyl-2,3,6,7-tetrahydro-1H-purin-8-yl)phenoxy)acetamido)ethyl)amino)-3-oxopropyl)carbamate [A, XAC- β-Ala-NHBoc]:

To a stirred solution of *N*-(2-aminoethyl)-2-[4-(2,6-dioxo-1,3-dipropyl-7H-purin-8-yl)phenoxy]acetamide (XAC, 100 mg, 0.233 mmol, 1.0 equiv., Tocris, Minneapolis, MN) and 4-(*N*,*N*-dimethylamino)pyridine (DMAP, 63 mg, 0.513 mmol, 2.2 equiv.) in DMF (2 mL), Boc-β-Ala-OH (44 mg,

0.233 mmol, 1.0 equiv.) was added, and the solution was stirred at room temperature (RT) for 5 min. 1-Ethyl-3-(3-dimethylaminopropyl)carbodiimide hydrochloride (EDC.HCl) (89 mg, 0.466 mmol, 2.0 equiv.) was added in one portion, and the reaction mixture was stirred at RT for two days. A white ppt was formed in the reaction mixture and the solution was diluted with water (20 mL). Thus, the obtained white ppt (**A, XAC-β-Ala-NHBoc**) was filtered off, washed with diethyl ether (5 mL), dried under high vacuum, and used without further purification for the next step. Yield: 45 mg, 32%.

^{1}H NMR (400 MHz, DMSO-d_6) δ 12.16 (s, 1H), 8.20 (t, J = 5.4 Hz, 1H), 8.09 (d, J = 8.7 Hz, 2H), 7.95 (t, J = 5.0 Hz, 1H), 7.10 (d, J = 8.8 Hz, 2H), 6.74 (br, 1H), 4.55 (s, 2H), 4.02 (t, J = 7.1 Hz, 2H), 3.87 (t, J = 7.8 Hz, 2H), 3.20 – 3.10 (m, 6H), 2.21 (t, J = 7.3 Hz, 2H), 1.77-1.72 (m, 2H), 1.62-1.56 (m, 2H), 1.36 (s, 9H), 0.93-0.86 (m, 6H).

HRMS: Cal. $C_{29}H_{42}N_7O_7$; 600.3146; found 600.3148(M + H)$^+$.

Step-2:

***tert*-Butyl (5-((3-((2-(2-(4-(2,6-dioxo-1,3-dipropyl-2,3,6,7-tetrahydro-1H-purin-8-yl)phenoxy)acetamido)ethyl)amino)-3-oxopropyl)amino)-5-oxopentyl)carbamate [B, XAC- β-Ala-Val-5-NHBoc]:**

Compound **A** (45 mg**,** 0.075 mmol, 1.0 equiv.) from Step-1 was dissolved in 1.0 mL of trifluoroacetic acid (TFA) and stirred at RT for overnight. TFA was removed by rotary evaporation, co-evaporated with toluene (2x3 mL), and dried under high vacuum to get the corresponding XAC-β-Ala.TFA salt. To a stirred solution of XAC-β-Ala.TFA salt (73 mg, 0.122 mmol, 1.0 equiv.) and DMAP (52 mg, 0.427 mmol, 3.5 equiv.) in DMF (2 mL), Boc-5-amino-valeric acid (37 mg, 0.171 mmol, 1.4 equiv.) was added and the solution was stirred at room RT for 5 min. EDC.HCl (47 mg, 0.244 mmol, 2.0 equiv.) was added in one portion, and the reaction mixture was stirred at RT for 16 h. The solvent (DMF) was removed by rotary evaporation to obtain a residue, which was dissolved in a mixture of MeOH and water (50 mL, 1:1) and filtered. The filtrate was kept at 0°C for 1 h to get a white ppt. Thus, the white ppt [**B**, **XAC- β-Ala-Val-5-NHBoc**] was filtered off, washed with diethyl ether (5 mL), and used without further purification for the next step. Yield: 15 mg, 18%.

^{1}H NMR (400 MHz, chloroform-d with a few drops of MeOH-d_4) δ 7.93 (d, J = 7.5 Hz, 2H), 7.28 (t, J = 3.4 Hz, 1H), 6.99 (t, J = 7.8 Hz, 2H), 4.48 (s, 2H), 4.07-4.06 (m, 2H), 3.93-3.89 (m, 2H), 3.35-3.28 (m, 4H), 3.03-2.97 (m, 4H), 2.17-2.14 (m, 2H), 2.09-2.07 (m, 2H), 1.79-1.75 (m, 2H), 1.64-1.54 (m, 6H), 1.37 (s, 9H), 0.94-0.87 (m, 6H).

HRMS: Cal. $C_{34}H_{51}N_8O_8$; 699.3830 m/z; found 699.3840 (M + H)$^+$

Step-3:

***tert*-Butyl (5-((3-((2-(2-(4-(2,6-dioxo-1,3-dipropyl-2,3,6,7-tetrahydro-1H-purin-8-yl)phenoxy)acetamido)ethyl)amino)-3-oxopropyl)amino)-5-oxopentyl)carbamate [C, XAC-β-Ala-Val-5-NH-6(Boc-NH)-Caprioc acid]:**

Compound **B** (33 mg, 0.0472 mmol, 1.0 equiv.) was dissolved in 1.0 mL of TFA and stirred at RT for overnight. The solution was concentrated by rotary evaporation, co-evaporated the residue with toluene (2x3 mL), and dried under high vacuum to get the corresponding XAC-β-Ala-Val-5-NH_2.TFA salt.

To a stirred solution of XAC-β-Ala-Val-5-NH_2.TFA salt (33 mg, 0.0472 mmol, 1.0 equiv.) and DMAP (14 mg, 0.118 mmol, 2.5 equiv.) in DMF (2 mL), Boc-6-amino-Caprioc acid (13 mg, 0.057 mmol, 1.2 equiv.) was added and the solution was stirred at RT for 5 min. EDC.HCl (18 mg, 0.0944 mmol, 2.0 equiv.) was added in one portion. After stirring the reaction mixture at RT for 16h, the solution was diluted with water (10 mL). A white ppt was formed in the reaction mixture, which was filtered off and purified by silica gel column to get the pure product [**C**, **XAC- β-Ala-Val-5-NH-6(Boc-NH)-Caproic acid**]. Eluant: 5-7% MeOH in DCM. Yield: 11.50 mg, 30%.

^1H NMR (400 MHz, chloroform-d with a few drops of MeOH-d_4): δ 7.98-7.92 (m, 2H), 7.30 (br, 1H), 7.04-6.97 (m, 2H), 4.49 (s, 2H), 4.07-4.04 (m, 2H), 3.93-3.89 (m, 2H), 3.38-3.31 (m, 6H), 3.10-3.08 (m, 2H), 2.99-2.97 (m, 2H), 2.29-2.28 (m, 2H), 2.10-2.08 (m, 4H), 1.79-1.74 (m, 2H), 1.64-1.61 (m, 2H), 1.59-1.51 (m, 4H), 1.39-1.35 (s, 12H), 1.21-1.17 (m, 3H), 0.96-0.87 (m, 6H).

HRMS: Cal. $C_{40}H_{62}N_9O_9$; 812.4670 m/z; found 812.4659 $(M + H)^+$

Step-4:

1-(4-(2,6-Dioxo-1,3-dipropyl-2,3,6,7-tetrahydro-1H-purin-8-yl)phenoxy)-2,7,11,17-tetraoxo-3,6,10,16-tetraazadocosan-22-aminium (XAC-245.TFA):

Compound **C** (11 mg, 0.0135 mmol) was dissolved in 1.0 mL of TFA and stirred at RT for overnight. The solution was concentrated by rotary evaporation, co-evaporated the residue with toluene (2x3 mL) and dried under high vacuum for overnight to get the compound **XAC-245** as a TFA salt (11 mg).
